# Supplementary material for: HIVprotI: an integrated web based platform for prediction and design of HIV proteins inhibitors
Source: J Cheminform. 2018 Mar 9;10:12. doi: 10.1186/s13321-018-0266-y (PMC5845081; doi:10.1186/s13321-018-0266-y)
Supplement: Supplementary file 2 — Additional file 2. Source code of HIVProtI web server. [file 13321_2018_266_MOESM2_ESM.zip › HIVprotI_Source-code/hivprotI/weka-3-6-6/doc/weka/core/GlobalInfoJavadoc.html]

GlobalInfoJavadoc


---


|  |  |  |
| --- | --- | --- |
| **Overview** \| **Package** \| **Class** \| **Tree** \| **Deprecated** \| **Index** \| **Help** \| **Weka's home** | |  |
| **PREV CLASS**   **NEXT CLASS** | **FRAMES**    **NO FRAMES**     **All Classes** |  |
| SUMMARY: NESTED \| FIELD \| CONSTR \| METHOD | DETAIL: FIELD \| CONSTR \| METHOD |  |


---


## weka.core Class GlobalInfoJavadoc

```
java.lang.Object
  weka.core.Javadoc
      weka.core.GlobalInfoJavadoc
```

**All Implemented Interfaces:**: OptionHandler, RevisionHandler

---

``` public class GlobalInfoJavadoc extends Javadoc ```

Generates Javadoc comments from the class's globalInfo method. Can
automatically update the comments if they're surrounded by
the GLOBALINFO\_STARTTAG and GLOBALINFO\_ENDTAG (the indention is determined via
the GLOBALINFO\_STARTTAG).


Valid options are:

```
 -W <classname>
  The class to load.
```

```
 -nostars
  Suppresses the '*' in the Javadoc.
```

```
 -dir <dir>
  The directory above the package hierarchy of the class.
```

```
 -silent
  Suppresses printing in the console.
```

**Version:**
:   $Revision: 1.7 $

**Author:**
:   fracpete (fracpete at waikato dot ac dot nz)

**See Also:**: `GLOBALINFO_METHOD`, `GLOBALINFO_STARTTAG`, `GLOBALINFO_ENDTAG`

---

| **Field Summary** | |
| --- | --- |
| `static java.lang.String` | `GLOBALINFO_ENDTAG`             the end comment tag for inserting the generated Javadoc |
| `static java.lang.String` | `GLOBALINFO_METHOD`             the globalInfo method name |
| `static java.lang.String` | `GLOBALINFO_STARTTAG`             the start comment tag for inserting the generated Javadoc |


**Constructor Summary**

`GlobalInfoJavadoc()`
  
          default constructor


| **Method Summary** | |
| --- | --- |
| `java.lang.String` | `getRevision()`             Returns the revision string. |
| `static void` | `main(java.lang.String[] args)`             Parses the given commandline parameters and generates the Javadoc. |

**Methods inherited from class weka.core.Javadoc**

`generate, generateHelp, getClassname, getDir, getOptions, getSilent, getUseStars, listOptions, setClassname, setDir, setOptions, setSilent, setUseStars, updateJavadoc`

**Methods inherited from class java.lang.Object**

`equals, getClass, hashCode, notify, notifyAll, toString, wait, wait, wait`

**Field Detail**

### GLOBALINFO\_METHOD

```
public static final java.lang.String GLOBALINFO_METHOD
```

:   the globalInfo method name

    **See Also:**: Constant Field Values

---


### GLOBALINFO\_STARTTAG

```
public static final java.lang.String GLOBALINFO_STARTTAG
```

:   the start comment tag for inserting the generated Javadoc

    **See Also:**: Constant Field Values

---


### GLOBALINFO\_ENDTAG

```
public static final java.lang.String GLOBALINFO_ENDTAG
```

:   the end comment tag for inserting the generated Javadoc

    **See Also:**: Constant Field Values


**Constructor Detail**

### GlobalInfoJavadoc

```
public GlobalInfoJavadoc()
```

:   default constructor


**Method Detail**

### getRevision

```
public java.lang.String getRevision()
```

:   Returns the revision string.

    :   **Returns:**: the revision

---


### main

```
public static void main(java.lang.String[] args)
```

:   Parses the given commandline parameters and generates the Javadoc.

    :   **Parameters:**: `args` - the commandline parameters for the object


---


|  |  |  |
| --- | --- | --- |
| **Overview** \| **Package** \| **Class** \| **Tree** \| **Deprecated** \| **Index** \| **Help** \| **Weka's home** | |  |
| **PREV CLASS**   **NEXT CLASS** | **FRAMES**    **NO FRAMES**     **All Classes** |  |
| SUMMARY: NESTED \| FIELD \| CONSTR \| METHOD | DETAIL: FIELD \| CONSTR \| METHOD |  |


---
